# Phylogenetic Analysis of *Hepatitis B* Virus among Household Members with HBV Chronic Infection

**Published:** 2019

**Authors:** Shahnaz Sali, Shirin Azarmmanesh, Hediyeh Ghalikhani, Maryam Vaezjalali

**Affiliations:** 1. Department of Infectious Diseases and Tropical Medicine, Shahid Beheshti University of Medical Sciences, Tehran, Iran; 2. Department of Immunology, Faculty of Medicine, Shahid Beheshti University of Medical Sciences, Tehran, Iran; 3. Department of Microbiology, Faculty of Medicine, Shahid Beheshti University of Medical Sciences, Tehran, Iran

**Keywords:** Genotype, Hepatitis B virus, Phylogenetic analysis, Surface antigen, Transmission

## Abstract

**Background:**

Intrafamilial spread of *Hepatitis B* virus (HBV) infection in Iran has only been investigated with serological testing without using molecular studies as the most informative and definitive type of analysis.

**Methods:**

In the present study, intrafamilial transmission of HBV among family members of Iranian index HBsAg carriers was investigated using phylogenetic analysis of the S region of the viral genome. Nested polymerase chain reaction was used for detection of HBV DNA in serum samples from 22 index and 43 contact patients with chronic HBV infection. HBV DNA was detected in 37 samples (14 indexes, 23 contacts). The S gene region of the DNA isolates was subjected to direct sequencing and phylogenetic analysis by Bioedit, Mega and Phylip programs.

**Results:**

All isolates (from 26 patients) were clustered with genotype D, of which 24 strains were of subgenotype D1, subtype *ayw2*, while 2 additional strains were of subgenotype D2, subtype *ayw3*. Evidence of intrafamilial transmission of the virus was found in 8 families studied phylogenetically. Overall, 60 changes were detected in the amino acid sequences of the surface antigen protein in 23 patients. Four premature stop codons occurred in 3 isolates at residues 69 and 182. Seven out of 8 families displayed 25–100% common amino acid substitutions among their members.

**Conclusion:**

Our data corroborated intrafamilial transmission of HBV, as evidenced by concordant HBV genotype among household members, viral sequence homology and close genetic relatedness of the strains on the phylogenetic tree, and horizontal transmission of S gene mutations among family members.

## Introduction

*Hepatitis B* virus (HBV) infection poses a major global health problem. Approximately, 2 billion individuals represent serological evidence of a current or prior HBV infection, from which about 400 million have a chronic hepatitis B infection 1. Globally, the predominant mode of HBV transmission is mother-to-child (or vertical) transmission, especially in regions with high carrier rates of the virus such as the Asia-Pacific region 2,3. Other modes of infection include parental and sexual transmission, as well as the intrafamilial spread of the virus. The presence of virus in saliva, urine and sweats of infected individuals has been documented and there is the possibility of viral spread by these routes through dermal or non-dermal pathways 4. Intrafamilial spread may occur through frequent and prolonged child-to-child or household contacts, probably *via* exposure to the bodily fluids of the infected person 5.

It has been shown that 35% of Iranian people had a history of HBV infection and approximately 2% of them were chronically infected. As there is no routine program for screening HBV infected individuals in Iran, there is a likelihood of infection transmission within family members in the undiagnosed period of HBV infection.

Intrafamilial HBV transmission has previously been investigated in a few Iranian studies using serological markers of infection 6,7. In these studies, family has been suggested as the focus of infection clustering without documenting it with molecular studies as the most appropriate method. Dumpis *et al* 8 documented HBV infection in Gambian children from family members and from other children in a village setting. Likewise, Lin *et al* 9 investigated the mode on familial transmission of HBV in a Taiwanese population by phylogenetic analysis and concluded that genotyping and phylogenetic analysis is highly useful in investigating intrafamilial spread of HBV virus. They reported that maternal and paternal transmissions were important routes of intrafamilial transmission in Taiwan.

In the present study, an attempt was made to confirm intrafamilial transmission of HBV among family members of Iranian index HBsAg carriers using phylogenetic tree analysis of nucleotide sequences generated by amplification of the S region of the virus genome.

## Materials and Methods

### Patients

During 2012–14, 22 index HBV infected individuals were recruited by convenient sampling from individuals who referred to the infectious disease clinic of a referral hospital who agreed to participate. Patients and their family members, who were HBsAg positive for at least 6 months, were included in this study. Patients with Human Immunodeficiency Virus (HIV), Hepatitis Delta Virus (HDV) or Hepatitis C Virus (HCV) and hepatocellular carcinoma were excluded.

Forty three family members of index patients with chronic HBV infection, who were positive for HBsAg, were also recruited for the study. There was no history of hemodialysis, blood transfusion, drug use, tattooing, or sexually transmitted diseases among the study patients. Written informed consent for participation in the study was obtained from all patients. This study was approved by the Ethics Committee of Shahid Beheshti University of Medical Sciences and is in accordance with the Helsinki declaration of 1964.

### DNA extraction and amplification

Five *ml* of venous blood was drawn from all patients (22 indexes and 43 contacts). DNA was extracted from 200 *μl* serum using phenol-chloroform method. Briefly, the serum was digested by proteinase K, followed by adding an equal volume of phenol-chloroform and vigorous vortexing. The organic and aqueous phases were separated by centrifugation at 12000 *g* for 15 *min* at 4*°C*, and the supernatant was collected. The process was repeated once more, followed by ethanol precipitation of nucleic acids. The pellet was resuspended in water and used for DNA amplification. The S region of the HBV genome was then amplified by nested Polymerase Chain Reaction (PCR) using specific primers. The resultant amplicon was separated by gel electrophoresis and visualized by ethidium bromide staining under UV transillumination.

### DNA sequencing and phylogenetic analysis

The amplicons were purified and subjected to bidirectional sequencing in an automated DNA sequencer (ABI Prism 3700 DNA Analyzer). Nucleotide sequence editing and alignment were performed using Chromas (Version 1.7.6) and Bioedit (Version 7.2.5) programs. A phylogenetic tree was constructed using the HBV S region nucleotide sequences isolated from the study patients (26 strains) and those obtained from the GenBank database (68 strains) at National Center for Biotechnology Information, NCBI. The construction and analysis of the phylogenetic tree were performed using Mega 6.0 and Phylip programs by neighbor-joining method. The phylogenetic tree was then used for genotyping, subgenotyping, and subtyping of the HBV DNA isolates.

## Results

### Overview of the patients

Overall, 65 patients (22 males, 43 females; mean age of 37.33 years) with chronic HBV infection were recruited. There were 22 index subjects and 43 contacts. The relationship of household contacts to index subjects included 7 parents (1 father, 6 mothers), 15 offspring (5 sons, 10 daughters), 14 siblings (5 brothers, 9 sisters), 3 wives, 2 grandchildren, 1 nephew, and 1 niece. The HBV DNA test was found positive in 37 patients (14 index cases, 23 contacts; mean age of 40.27 years) whose PCR products were subjected to direct sequencing. Twenty-eight additional patients did not yield any sequences (Table 1).

**Table 1.** Characteristics of the patients

**Family**	**Subject**	**Relation**	**Gender**	**Age**	**HBV DNA**	**Sequencing**	**Genotype**	**Subtype**	**S-gene product mutation**	**Mutation frequency**
**1**	1	Index	M	42	–					
	2	Spouse	F	37	–					

**2**	3	Index	F	52	–					
	4	Offspring	F	32	–					

**3**	5	Index	F	57	+	+	D1	*ayw2*	I208T	1
	6	Offspring	F	31	–					
	7	Grandchild	M	10	+	+	D1	*ayw2*	–	0
	8	Grandchild	F	5	–					

**4**	12	Index	M	58	+	+	D1	*ayw2*	G145R, I195M, S204N, Y206C, S210K, I213S, C221F	7
	9	Sibling	F	51	+	+	D1	*ayw2*	I195M, S204N, Y206C, S210K, I213S, C221F	6
	10	Niece	F	27	+	+	D1	*ayw2*	S210T	1
	11	Sibling	F	56	+	+	D1	*ayw2*	C69^*^, P105A, F134N, G145E, W182^*^	5
	13	Nephew	M	32	–					
	14	Offspring	F	26	–					
	44	Spouse	F	52	–					

**5**	15	Index	F	56	+	+	D1	*ayw2*	G44E, Y206C, S207N, I208T, P217L	5
	16	Offspring	F	35	–					

**6**	17	Index	M	21	+	–				
	18	Parent	F	45	–					

**7**	19	Index	M	18	–					
	20	Sibling	F	20	+	+	D1	*ayw2*	W182G	1

**8**	21	Index	F	31	+	+	D2	*ayw3*	P127T, S136Y	2
	22	Parent	F	52	+	+	D2	*ayw3*	P127T, S136Y	2

**9**	23	Index	F	27	+	+	D1	*ayw2*	–	0
	24	Parent	F	53	+	–				

**10**	25	Index	F	37	+	–				
	26	Sibling	F	30	+	–				
	27	Sibling	F	33	+	–				

**11**	30	Index	F	45	+	+	D1	*ayw2*	F8L	1
	28	Offspring	M	21	+	–				
	29	Offspring	M	18	–					

**12**	31	Index	F	29	+	+	D1	*ayw2*	T143L, S207R	2
	32	Parent	M	62	+	+	D1	*ayw2*	–	0
	33	Parent	F	55	+	+	D1	*ayw2*	C76Y, T143L	2
	34	Sibling	M	37	+	+	D1	*ayw2*	T143L, S204N	2
	35	Sibling	F	27	+	–				
	36	Sibling	M	22	+	+	D1	*ayw2*	R79H, T143L, S204N	3

**13**	37	Index	F	57	+	+	D1	*ayw2*	C69^*^	1
	38	Offspring	M	33	+	+	D1	*ayw2*	C69^*^, Y200N	2

**14**	47	Index	M	21	+	+	D1	*ayw2*	F8L	1
	46	Parent	F	46	–					
	48	Sibling	F	25	+	+	D1	*ayw2*	F8L	1

**15**	49	Index	F	60	+	+	D1	*ayw2*	F8P, C76Y, P120S, S207N	4
	50	Offspring	F	39	+	+	D1	*ayw2*	F8P, C76Y, P120S, S207N	4
	51	Offspring	F	47	+	+	D1	*ayw2*	F8P, S207N	2

**16**	52	Index	M	54	–					
	53	Sibling	M	59	–					

**17**	54	Index	M	36	–					
	55	Sibling	F	29	–					
	56	Sibling	F	18	–					

**18**	57	Index	F	22	–					
	58	Sibling	F	27	–					
	59	Sibling	M	31	–					

**19**	60	Index	F	41	+	–				
	61	Parent	F	70	+	–				

**20**	64	Index	M	35	–					
	65	Spouse	F	29	–					
	66	Offspring	F	10	–					

**21**	62	Index	M	40	–					
	63	Sibling	M	32	+	–				

**22**	70	Index	F	65	+	+	D1	*ayw2*	S207N	1
	67	Offspring	M	38	+	+	D1	*ayw2*	N40S, I86T, V96A, S207N	4
	68	Offspring	F	45	+	–				
	69	Offspring	M	41	–					
	71	Offspring	F	35	–					

Asterisks signify premature stop-codon; blank cells represent “Not available”. Patient 10 was the daughter of patient 9. Patient 13 was the son of patient 11.

### Phylogenetic analysis, genotyping and intrafamilial transmission

**Figure 1.**
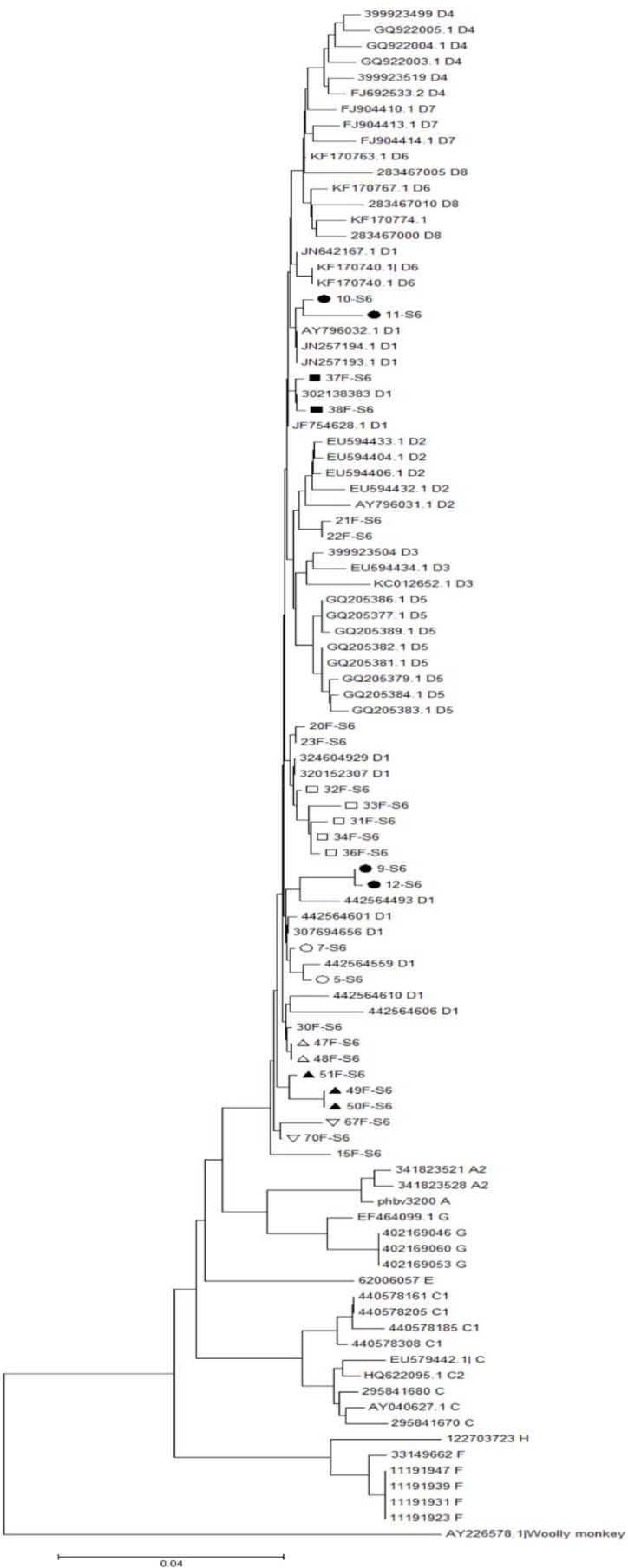
Phylogenetic tree of HBV S-gene partial sequence constructed using 26 isolates from this study along with 68 Genbank reference sequences. Samples from the present study are represented by the patient number plus S6 (*e.g*. F15-S6). Reference strains from GenBank database are designated by their accession number. Branch lengths are proportional to sequence divergence. Similar geometric shapes within a node denote that they belong to one family.

Partial S-gene sequencing (Nucleotides 1-681) and subsequent phylogenetic analysis were achieved in 26 (11 indexes, 15 contacts) out of 37 patients with positive PCR products. However, only 8 distinct families had at least one index and one contact subject with successful sequencing and phylogenetic analysis. A phylogenetic tree was constructed using the nucleotide sequences of the surface antigen protein gene obtained from the study patients (26 strains) and those that were available in the GenBank database (68 strains, Figure 1). Phylogenetic analysis revealed that all of the strains from the patients were clustered with genotype D, which is the predominant genotype in Iran. Specifically, 24 strains were of subgenotype D1, while 2 additional strains from the same family were of subgenotype D2. The isolates with subgenotype D2 were of subtype ayw3. The remaining isolates with subgenotype D1 were of subtype ayw2. Of the 8 families analyzed phylogenetically, 7 showed distinct grouping comprising all members from each family, confirming intrafamilial transmission of the virus. One additional family of four (Family 4) displayed two separate yet distinct grouping, each with two individuals, indicating the involvement of two different virus isolates.

### Mutations in the HBV S-gene

The nucleotide and predicted amino acid sequences of the HBV S-gene from the patients were compared with the reference sequence pHBV3200. Based on the primers selected for PCR amplification of the DNA isolates, it was possible to evaluate 227 amino acids in the HBsAg protein. The frequency and position of different mutations within the nucleotide sequences and the resultant amino acid changes within the surface antigen protein are summarized in table 2. Overall, 60 changes occurred in the amino acid sequences of 23 patients predicted by the observed point mutations in their DNA isolates. Three individuals (2 from the same family) carried premature termination codon at residue 69, leading to a truncated surface protein missing the “a” determinant (amino acids 124–147) and the major hydrophilic (MHR, amino acids 99–169) regions. One of these patients had an additional premature termination codon at residue 182. The most frequent variations in the surface protein amino acid sequences were S207N, S204N, and T143L, as they were detected in 6 (26.1%), 4 (17.4%), and 4 (17.4%) isolates, respectively. Moreover, 14 amino acid substitutions were clustered in the major hydrophilic region of HBsAg, of which 11 occurred in the “a” determinant region. Frequency of shared amino acid substitutions in the surface antigen protein among the family members is shown in table 3. Except one family (Family 3), the remaining 7 families had 25–100% common substitutions among their members, which further supports an intrafamilial mode of infection.

**Table 2.** The frequency and position of different point mutations within the HBV surface antigen sequences from the study patients

**Mutations in S region**	**Amino acid substitutions**	**Frequency of amino acid substitutions**	Proposed antigenic epitope^*^	**Amino acid sequence of the epitope**
**T22C**	F8L	3		
**T23C**	F8P	3		
**A119G**	N40S	1	T helper (CD4)	21–65
**G131A**	G44E	1	T helper (CD4)	21–65
**T207A**	C69^*^	3		
**G227A**	C76Y	3		
**G236A**	R79H	1		
**T257C**	I86T	1	T helper (CD4)	80–98
**T287C**	V96A	1	T helper (CD4)	80–98
**C313G**	P105A	1	B cell	100–160
**C358T**	P120S	2	B cell	100–160
**C379A**	P127T	2	B cell	100–160
**T400A**	F134N	1	B cell	100–160
**C407A**	S136Y	2	B cell	100–160
**C428T**	T143L	4	B cell	100–160
**G433A**	G145R	1	B cell	100–160
**G434A**	G145E	1	B cell	100–160
**T544G**	W182G	1	Cytotoxic T cell (CD8)	175–184
**G546A**	W182^*^	1		
**A585G**	I195M	2	T helper (CD4)	186–197
**T598A**	Y200N	1		
**G611A**	S204N	4		
**A617G**	Y206C	3	Cytotoxic T cell (CD8)	206–215
**A619C**	S207R	1	Cytotoxic T cell (CD8)	206–215
**G620A**	S207N	6	Cytotoxic T cell (CD8)	206–215
**T623C**	I208T	2	Cytotoxic T cell (CD8)	206–215
**G629A, T630G**	S210K	2	Cytotoxic T cell (CD8)	206–215
**G629C**	S210T	1	Cytotoxic T cell (CD8)	206–215
**T638C**	I213S	2	Cytotoxic T cell (CD8)	206–215
**C649T**	P217L	1	T helper (CD4)	215–223
**G662T**	C221F	2	T helper (CD4)	215–223

Asterisks signify premature stop codon.

* The proposed antigenic epitope information comes from previous studies by other researchers (32,33).

**Table 3.** Shared amino acid substitutions among family members with sequenced isolates

**Family**	**Frequency of patients**	**Patients with shared substitutions**	**Frequency of shared substitutions**	**Percentage of homology in amino acid substitutions**
3	2	–	–	–
4	4	12, 9	6 of 7	85.7
		10, 11	–	–
8	2	21, 22	2 of 2	100
12	5	31, 33, 34, 36	1 of 4	25
		34, 36	2 of 3	66.66
13	2	37, 38	1 of 1 (premature stop-codon)	100
14	2	47, 48	1 of 1	100
15	3	49, 50, 51	2 of 4	50
		49, 50	4 of 4	100
22	2	70, 67	1 of 4	25

Patients with underlined numbers are index cases.

## Discussion

The HBV infection’s prevalence in Iran has been estimated to be 2.14%. As a result, about 1.5 million people in Iran are thought to be living with HBV infection, which corresponds to mild to moderate prevalence according to WHO classification. It is also assumed that 15–40% of them are at risk of developing cirrhosis and/or hepatocellular carcinoma 10.

In the present study, 22 families of 2–7 members with chronic HBV infection were evaluated. According to the latest general population census in Iran in 2011, the average household size was 3.55. The average number of persons with HBV among the families studied here was 2.95, which shows clustering of HBV infection within these families. Viral transmission through intrafamilial contact is thought to critically contribute to the clustering of the HBV infection within family groups 5. The intrafamilial childhood horizontal transmission has been proposed as the predominant route by which the HBV endemicity rates are maintained in the Middle East. Additionally, this pattern of transmission persists into adult life 11. Intrafamilial clustering of viral infection has previously been shown in Iran 6,7,12. However, familial clustering does not necessarily conclude that intrafamilial spread is the cause of familial clustering. Infection of other family members can also happen as a result of similar risky behaviors or lack of hygiene within a family. In order to remove this ambiguity and report intrafamilial transmission as the route of infection spread, phylogenetic analysis should be resorted to.

Our results also indicate the circulation of the virus among parents (16.3%), offspring (34.9%), and siblings (32.5%) of the primary cases. In order to further confirm the intrafamilial transmission, phylogenetic analysis of nucleotide sequences amplified from the S region of the HBV genome was performed in 26 patients with chronic HBV infection. Sixty-eight strains from the GenBank database were also used to construct the phylogenetic tree. The results showed that all viral sequences from the patients under investigation were clustered with genotype D, the predominant genotype in Iran 13–18 and the Middle East 19. Of the 26 isolates analyzed, 24 were of subgenotype D1, subtype awy2, whereas the remaining 2 isolates from the same family were of subgenotype D2, subtype ayw3. This is consistent with previous reports that the strains from the Middle East mainly belonged to subgenotype D1 20. Indeed, subgenotype D1 is restricted to Iran and its neighboring countries, whereas D2 is derived from East Europe and Russia 21.

Another study conducted in North of Iran demonstrated that all viral DNA isolates from 100 patients evaluated belonged to genotype D, subgenotype D1, subtype ayw2 22. These observations may be of clinical impact, in that, compared to the genotype A, genotype D has been shown to have lower tendency of chronicity, lower rate of HBeAg positivity, later HBeAg seroconversion, less HBsAg seroclearance, higher histologic activity, worse clinical outcome, and lower response to interferon alpha 23.

While a concordant HBV genotype among household members may provide possible evidence of intrafamilial transmission, the infectious source of family members could not be confidently identified by genotyping data alone, especially in studies carried out in regions with extremely restricted distribution of the viral genotypes. Therefore, phylogenetic analysis of the viral genome is a useful approach to confirm precisely the source of HBV infection 24. As a matter of fact, phylogenetic analysis of viral DNA sequences has been used in several studies to reliably confirm intrafamilial transmission of HBV 9,25,26. In this regard, phylogenetic analysis provided strong evidence of intrafamilial transmission of the virus in families studied in Italy 27 and Gambia 8. Additionally, using viral sequence homology and detailed history, it has been revealed that horizontal mode of transmission and a common source of infection were frequent among household members with HBV-related chronic liver disease in selected Indian families 28. In line with the aforementioned studies, phylogenetic tree analysis of DNA fragments replicated from the S region of the HBV genome revealed that patients from the same families had very closely related isolates. Indeed, from 8 families investigated phylogenetically, members of 7 families grouped together on separate branches of the tree, indicating the close genetic relatedness of the virus isolates within the families and their common source of infection. In the remaining family (Family 4), however, two members (the index case and one of his sisters) were located on one branch, and other two members (Another sister and the niece) were laid on a much farther branch, suggesting two different patterns of intrafamilial transmission with different sources of infection.

Additional observation that supports intrafamilial transmission of the virus among the patients studied is that similar mutations in the S-gene were transmitted horizontally. Our results showed that in 7 out of 8 families evaluated, there was 25–100% homology in amino acid substitutions occurred within the surface protein. A previous report indicated that HBsAg mutant G145R, the most common HBsAg mutant which shows the highest incidence in both the vaccinated and random populations, could be transmitted horizontally among family members 29.

Mutations in the S-gene, which codes for HBsAg 30, especially those occurring in the “a” determinant in the major hydrophilic loop, can influence virion secretion and decrease binding of HBsAg to anti-HBs antibodies 31. Consequently, they may alter viral antigenicity, thereby resulting in escape to vaccination-induced neutralizing antibodies and interference with the diagnostic assays for detection of HBsAg 29. Of particular note, 11 amino acid changes (19.6%) were identified in the “a” determinant region. This HBV antigenic region, which encompasses residue 124–147 on HBsAg, is involved in inducing protective antibodies in an infected host.

## Conclusion

In conclusion, our data corroborated intrafamilial transmission of HBV, as evidenced by the concordant HBV genotype among household members, viral sequence homology and close genetic relatedness of the strains on the phylogenetic tree, and horizontal transmission of S gene mutations among family members.
